# Random lasing and amplified spontaneous emission from silk inverse opals: Optical gain enhancement via protein scatterers

**DOI:** 10.1038/s41598-019-52706-4

**Published:** 2019-11-07

**Authors:** Muhammad Umar, Kyungtaek Min, Sookyoung Kim, Sunghwan Kim

**Affiliations:** 1 Department of Energy Systems Research, Ajou University, Suwon, 16499 Republic of Korea; 2 Department of Nano-Optical Engineering, Korea Polytechnic University, Siheung, 15073 Republic of Korea; 3 Department of Physics, Ajou University, Suwon, 16499 Republic of Korea

**Keywords:** Optical materials and structures, Materials science

## Abstract

Gain amplification and coherent lasing lines through random lasing (RL) can be produced by a random distribution of scatterers in a gain medium. If these amplified light sources can be seamlessly integrated into biological systems, they can have useful bio-optical applications, such as highly accurate sensing and high-resolution imaging. In this paper, a fully biocompatible light source showing RL and amplified spontaneous emission (ASE) with a reduced threshold is reported. Random cavities were induced in a biocompatible silk protein film by incorporating an inverse opal with an inherent disorder and a biocompatible dye for optical gain into the film. By choosing the appropriate air-sphere diameters, clear RL spikes in the emission spectra that were clearly distinguished from those of the ASE were observed in the silk inverse opal (SIO) with optical gain. Additionally, the RL output exhibited spatial coherence; however, the ASE did not. The high surface-to-volume ratio and amplification of the SIO led to highly efficient chemosensing in the detection of hydrogen chloride vapor. Moreover, SIO could be miniaturized to be made suitable for injection into biological tissues and obtain RL signals. Our results, which open the way for the development of a new generation of miniaturized bio-lasers, may be considered as the first example of engineered RL with biocompatible materials.

## Introduction

Biomaterial-based optical devices have utility in various healthcare applications, such as sensing, imaging, and therapy, because of their versatile functionalities; their bio-friendly characteristics make their integration with living tissue feasible^1–3^. Among numerous biomaterials, silk fibroin, the natural protein that can be extracted from the *Bombyx mori* caterpillar, is gaining an increasingly prominent role as an optical matrix owing to its remarkable transparency, biocompatibility, and easy nanostructuring and functionalization^4–6^. Various silk-based optical devices have been realized, such as metal–insulator–metal resonators, metamaterials, gratings, and lasers^7–13^. In addition, silk fibroin has been reported as a biocompatible polymeric material^14–17^, therefore a favorable combination of silk protein and non-toxic fluorescent organic dyes leads to fully biocompatible photonic devices for biological and biomedical applications.

Especially, compact and highly coherent biological light sources are highly desirable because high performance and an expanded application sphere can be obtained in healthcare applications^18,19^. As such, investigations regarding the emission of active materials embedded in biomaterial-based resonator structures have been conducted. Various biological lasers using conventional lasing schemes, such as planar microcavities, microspheres, and distributed feedback structures^20–23^, have been devised. Several studies have reported random lasing (RL) from self-assembled biomaterial nanostructures^24–26^. However, it is still arguable whether the resulting broad spectral peaks originate from the stimulated emission. Since a number of papers have been reported on RL, it is very important to verify whether the optical signals obtained from the reported RLs satisfied the fundamental properties of the laser, such as narrow spectral peaks and coherences.

The pursuit of coherent lasing, fully biological devices, easy fabrication, and low cost is attracting attention toward self-assembled three-dimensional photonic crystals (PhCs), which are also known as opals, prepared through the self-assembly of colloidal crystals and their derivatives^27–29^. In a PhC, a propagating group of photons can be reflected or slowed down owing to coherent feedback by periodic scatterers^29^. Additionally, photons can be spatially localized by inherent or artificially generated disorder in the fabrication of a PhC. RL can occur when the disordered structure contains optical gain and the gain forms sufficiently strong stimulated emissions before the amplified light escapes the structure owing to scattering^30,31^. If the quality (Q) factor of a resonant cavity is not sufficient to induce lasing, then the reduction of the amplified spontaneous emission (ASE) threshold or enhancement of fluorescence are possible^32^.

## Results and Discussions

Here, we report a bio-friendly light source that comprises silk protein and fluorescein sodium, which is a biocompatible dye with diagnostic applications. This source reveals narrow RL lines superimposed on the ASE band along with a reduced ASE threshold. Silk inverse opals (SIOs) with optical gain were generated and optically pumped to investigate their luminescent properties. Random cavities existed in the SIOs owing to the refractive index (RI) inhomogeneity caused by structural imperfection during fabrication, and these induced a gain enhancement that led to a reduction in the ASE threshold and RLs. The shorter the diameter of air voids was, the easier we could obtain RLs, indicating that more high-Q random cavities were formed. Moreover, the orderedness of the SIO did not have a significant effect on lasing. RLs showed spatial coherence in Young’s double-slit experiment, whereas ASE did not. The large surface-to-volume ratio and gain-enhancement mechanism in SIOs were useful for obtaining a very high sensitivity in the chemosensing process. In addition, sub-mm SIO particles were generated; they exhibited ASE and RL when the particles were integrated underneath the cover tissue of an onion.

**Figure 1:**
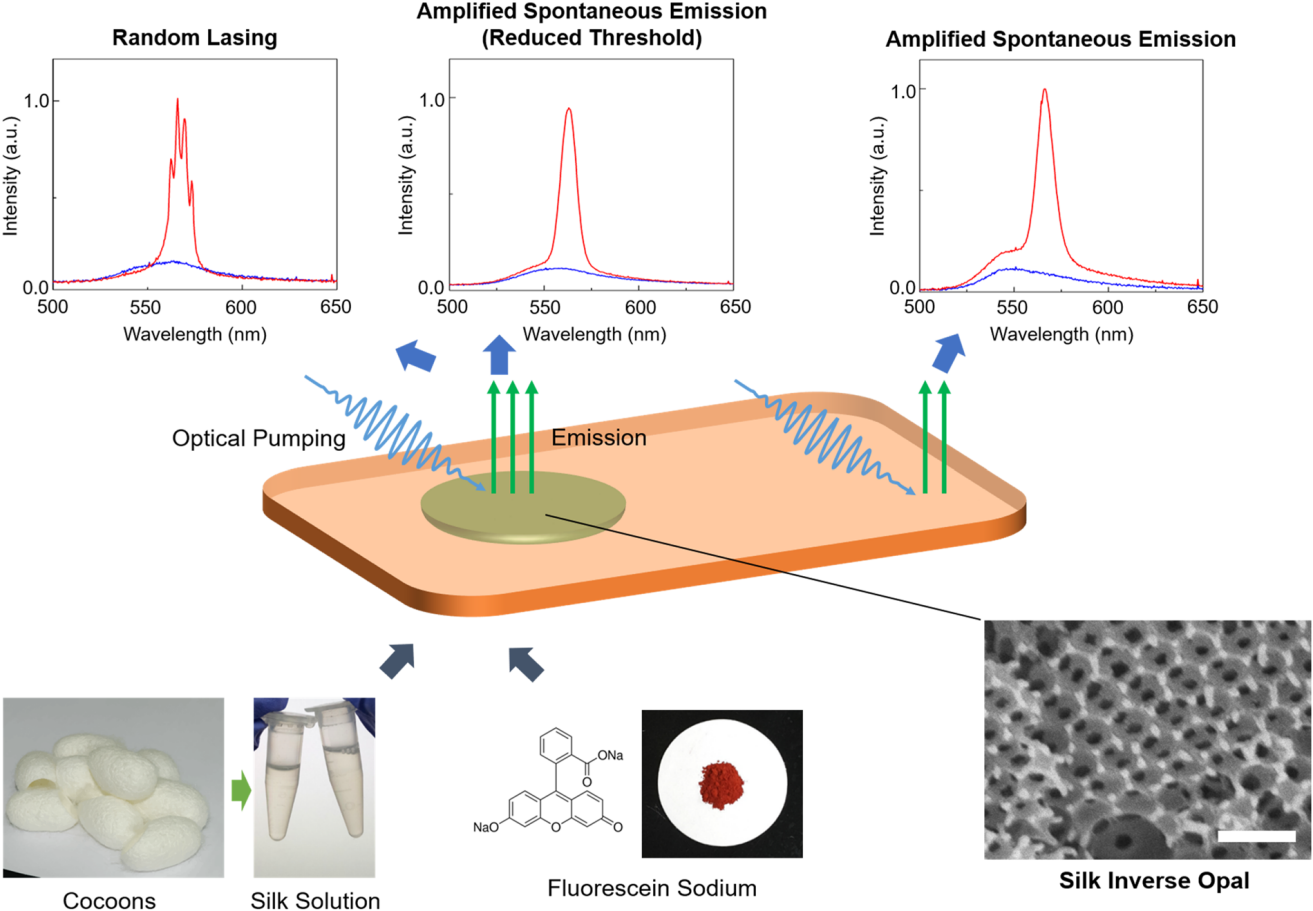
RL and ASE using the silk protein opal. Schematic showing that the integration of an inverse opal to a silk film with optical gain can induce RL or reduce the threshold of ASE. The same spectrum of ASE can be observed from the bulk silk film. For most area of the SIO, ASEs with reduced thresholds are observed. However, the RL can be only obtained when the locally disordered area yields a resonator with a high-quality (Q) factor.

SIOs can be integrated into a free-standing silk film through the selective wet etching of poly(methyl methacrylate) (PMMA) spheres used as a template (Fig. 1)^29^. As shown in the scanning electron microscopy image, periodic air voids were generated in the silk film. However, because of the imperfections in fabrication, disorders such as air voids with different sizes and cracks could result. The optically transparent silk matrix turned into a gain medium after dye molecules were added to the silk solution before casting. The third harmonic of a pulsed neodymium-doped yttrium aluminum garnet (Nd:YAG) laser at 355 nm (pulse duration of 25 ps and repetition ratio of 2 Hz) pumped the dye-doped silk film and the SIO to exhibit ASE and RL. In refs^24,25^, the authors claim that the spectra were obtained from similar structures to our SIO show RL and that they engineered the first silk-based biocompatible RL However, the narrowed spectral peak above threshold with about 10-nm full width at half maximum (FWHM) just indicated the transition to the ASE from the photoluminescence (PL, the fluorescence of a dye). Further, we confirmed that our bulk silk/dye film with no micro/nanostructures exhibited the same spectral behaviors as those reported in refs^24,25^. Our investigation revealed that the incorporation of micro/nanoscatterers could reduce the threshold of ASE, and the real RL could arise when the inhomogeneity of the RI distribution yielded a high-Q random cavity.

**Figure 2:**
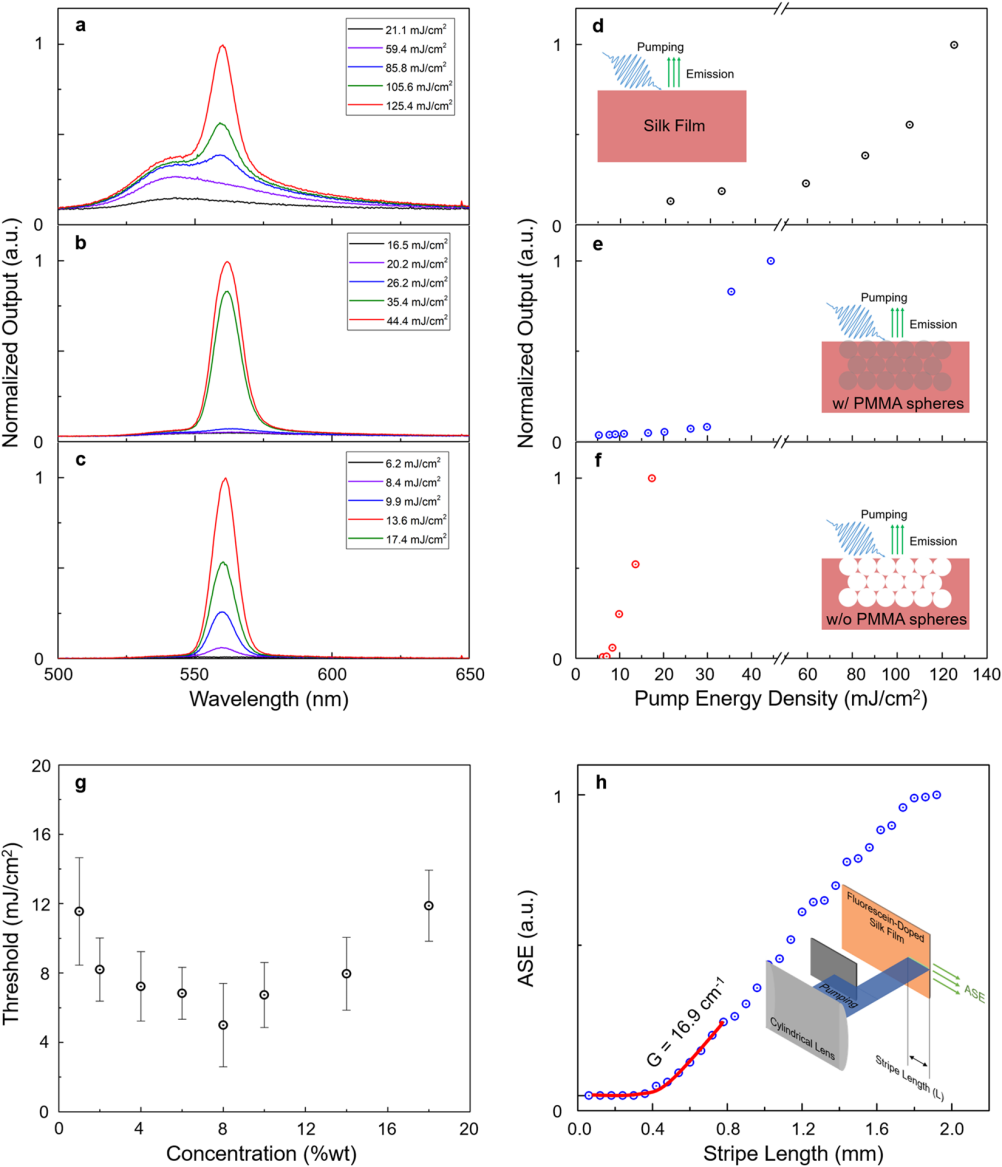
Optical gain and ASE spectra from silk/dye films. (**a**–**f**), Pump-fluence dependences of the ASE emissions and threshold behaviors on the bulk silk/dye film: (**a**,**d**), the silk/dye film with the PMMA opal (**b**,**e**), and the silk/dye film with air voids after removing the PMMA opal (**c**,**f**). The concentration of the sodium fluorescein dye was 10 wt%. As RI contrast (Δ*n*) of the opal structure was increased, the threshold was greatly reduced owing to the increased optical path. (**g**) The measured threshold pumping power of ASEs from the bulk dye/silk film with various concentrations of the sodium fluorescein dye. The error bar represents the standard deviation calculated using five data points at each concentration. (**h**) The variable stripe-length experiment to estimate the net modal gain of the bulk silk film mixed with sodium fluorescein. The concentration of sodium fluorescein was 8 wt%, and all spectra were excited at *λ* = 355 nm with a 25-ps pulse width.

Increasing the light scattering induces the shortening of the mean free path of the generated light. As shown in Fig. 2a–f, ASEs were observed for the following three states: bulk, incorporation of a PMMA opal, and an SIO (after removing the PMMA spheres). The concentration of sodium fluorescein was 10 wt%, and the radius of the PMMA spheres was 300 nm. Interestingly, when the weak scatterer with the RI contrast (Δ*n*) of 0.06 (for the sample with the PMMA opal, *n*_silk_ = 1.54 and *n*_PMMA_ = 1.48) was exhibited, the threshold of ASE was reduced from ~80 to ~30 mJ/cm^2^. Furthermore, the selective removal of the PMMA spheres (yielding the SIO) increased the strength of the scattering by the enlarged Δ*n* of 0.54. This could reveal the dramatically reduced ASE threshold to ~7 mJ/cm^2^, which is more than 10 times less than that of the bulk. Additionally, the ASE phenomena could be observed when the different gain dye (Rhodamine B) was used in the SIO (Supplementary Fig. 1). Above the threshold, the crossover from fluorescence to ASE was observed; this result is the same as that shown in ref^24^. One dignifies the transition to ASE with diffusive or incoherent RL with nonresonant feedback. Here we would like to provide our RL experimental results and theoretical analysis in a reliable way to prove that our implementation is definitely a “laser”^33^.

Figure 2g shows the thresholds of building ASE for varying concentrations of sodium fluorescein dye. As the concentration increases up to 8 wt%, the ASE threshold decreases. However, at the higher concentration, the threshold starts increasing because of the intermolecular quenching by the aggregated dye molecules^34^. This result indicates that the concentration must be kept to ~8 wt% to obtain highly efficient spontaneous emission. In addition to the threshold, the narrower ASE spectrum was red-shifted by approximately 15 nm with respect to the fluorescence maximum. When the ASE and fluorescence spectra are overlaid with the replotted absorption using Tauc’s method (Supplementary Fig. 2), the ASE spectral maximum coincides with the end of the shallow absorption tail (Urbach tail) and indicates the direct band gap absorption of the sodium fluorescein dye at ~2.33 eV^35^. The red-shifted ASE may have its origin in reabsorption due to the overlap of the onset between absorption and emission or the fast nonradiative decay taking place in the higher energy region^36^. Although a very high pumping power (~1 J/cm^2^) was applied, the ASE could disappear under 10-ns pulse pumping (Supplementary Fig. 3). This result indicates that the system enters a quasi-continuous-wave regime of excitation under the ns pumping, and the ASE lifetime is much shorter than the ns level^37^. Additionally, we investigated the net modal gain, which is an important figure of merit that indicates the efficiency of amplification and quality of the resonator for the achieving laser^37,38^. As described in the inset of Fig. 2h, the dye/silk film was excited with a line-shaped pumping light with variable lengths. Then, the emission intensity was measured as a function of stripe length *L*. At the threshold length, the optical amplification compensated for the propagation loss, and the PL spectrum started to reveal the ASE. The threshold region was fitted with the model of net modal gain *G*: $I=\frac{A}{g}({e}^{{GL}}-1)$. We obtained 16.9 cm^−1^ as the value of *G*, which is a moderate value in optical systems containing organic dyes.

**Figure 3:**
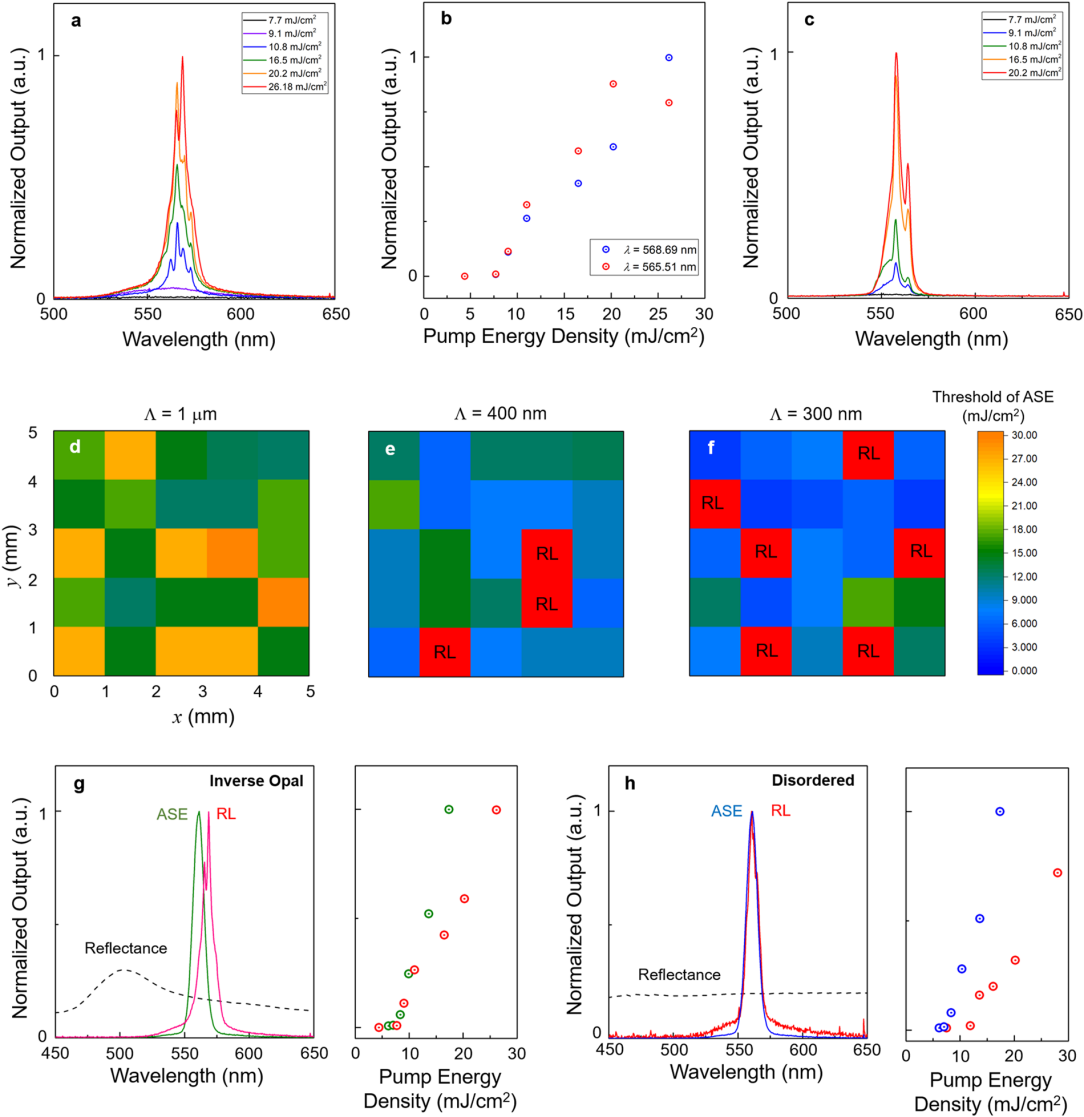
RLs from the SIOs. (**a**) Evolution from PL to RL with increasing pump intensity in the SIO. (**b**) The threshold behaviors of two RL modes shown in (**a**,**c**) RL spectra measured on different pumping spots of the same SIO in **a**. (**d**–**f**) ASE threshold distributions from the SIOs with different sizes of air voids (Λ = 1, 0.4, and 0.3 μm). As the size of air voids was decreased, the ASE threshold was reduced, and it was easier to obtain RLs. (**g**,**h**) Spectra and light-in versus light-out (LL) curves of ASE and RL from the ordered and disordered SIOs. All the samples were made using PMMA spheres with a 300-nm diameter. The reflectance spectra were merged in the ASE and RL spectra plots to show the existence of the photonic band gap related to the orderness of the SIO.

Narrow RL lines superimposed on the ASE band appeared when the pumping spot moved and coincidentally reached the random cavity with a sufficient Q factor, as shown in Fig. 3a^30,39,40^. Above the threshold, we observed 4 distinct peaks with an average FWHM of 1.8 nm (close to the resolution limit of the used spectrometer) and mode spacing Δ*λ* around 3.5 nm. Threshold energies for all RL modes were similar to those for ASEs (~8 mJ/cm^2^); however, the slope efficiencies were different (Fig. 3b) because of mode competition. On an SIO, we observed several points showing RL (Fig. 3c). The FWHM of all RL modes had similar values of ~1.8 nm; however, the number of modes was limited to four or five modes because the ASE band showing high optical gain was approximately 15 nm and the Δ*λ* was more than 3 nm. In addition, the Δ*λ* can tell us the estimated cavity round-trip length *L*_c_ by the equation *L*_c_ = *λ*2/(*n*Δ*λ*), where *n* is the RI and *dn*/*dλ* is neglected^41^. We obtained an estimated *L*_c_ of 60 μm for the SIO-based RL. The Q factor, sufficient to lase, of the local random cavity could worsen when the RI contrast (Δ*n*) was reduced. It was observed that the RL disappeared (reappeared) when the SIO was immersed (dried) in isopropyl alcohol (IPA) (Supplementary Fig. 4).

In the case of inverse opals, the strength of scattering depends on the size of air voids because the density of scatterers is fixed. Efficient scattering is obtained when the size of scatterers is comparable with the wavelength of light. Figures 3d–f show the two-dimensional mappings of ASE thresholds for SIOs with different air-void diameters (1.0, 0.4, and 0.3 μm). Areas sized 5 × 5 mm^2^ with a 1-mm step for all the SIOs were scanned by moving the sample state. All the excited spots have different Q factors for the random cavity; therefore, the ASE thresholds have different values. As the diameter of voids is decreased, the ASE threshold tends to decrease and the number of RL spots increases, indicating that random cavities with high-Q factors arise because of the shortened scatterers. It is noteworthy that we observed no RL behavior at the 1-μm SIO for which refs^24,25^ reported gain enhancement, but RLs were obtained even when the diameter was reduced to 100 nm (Supplementary Fig. 5). In addition, no RLs were raised under the 50-nm diameter because the light wave could feel no scattering (data not shown). Another arguable point is the effects of the PhC on RL, such as gain enhancement by the photonic band edge^31,32^. Figure 3g shows the spectra and the light-in versus light-out curves of ASE and RL signals from the ordered SIO (Λ = 300 nm), along with the reflectance spectrum showing the photonic band gap at the 500 nm wavelength. The gain-enhancement effect owing to the photonic band edge on the ASE and RL signals may be expected. However, the same ASE and RL signals with similar thresholds could be observed from the totally disordered SIO (no band gap), as shown in Fig. 3h, indicating that the only local RI inhomogeneity contributed to yielding a high-Q random cavity. Additionally, RL and ASE signals were obtained for the same structures (disordered and ordered SIOs), when stilbene dye showing blue emission was applied, (Supplementary Fig. 6).

**Figure 4:**
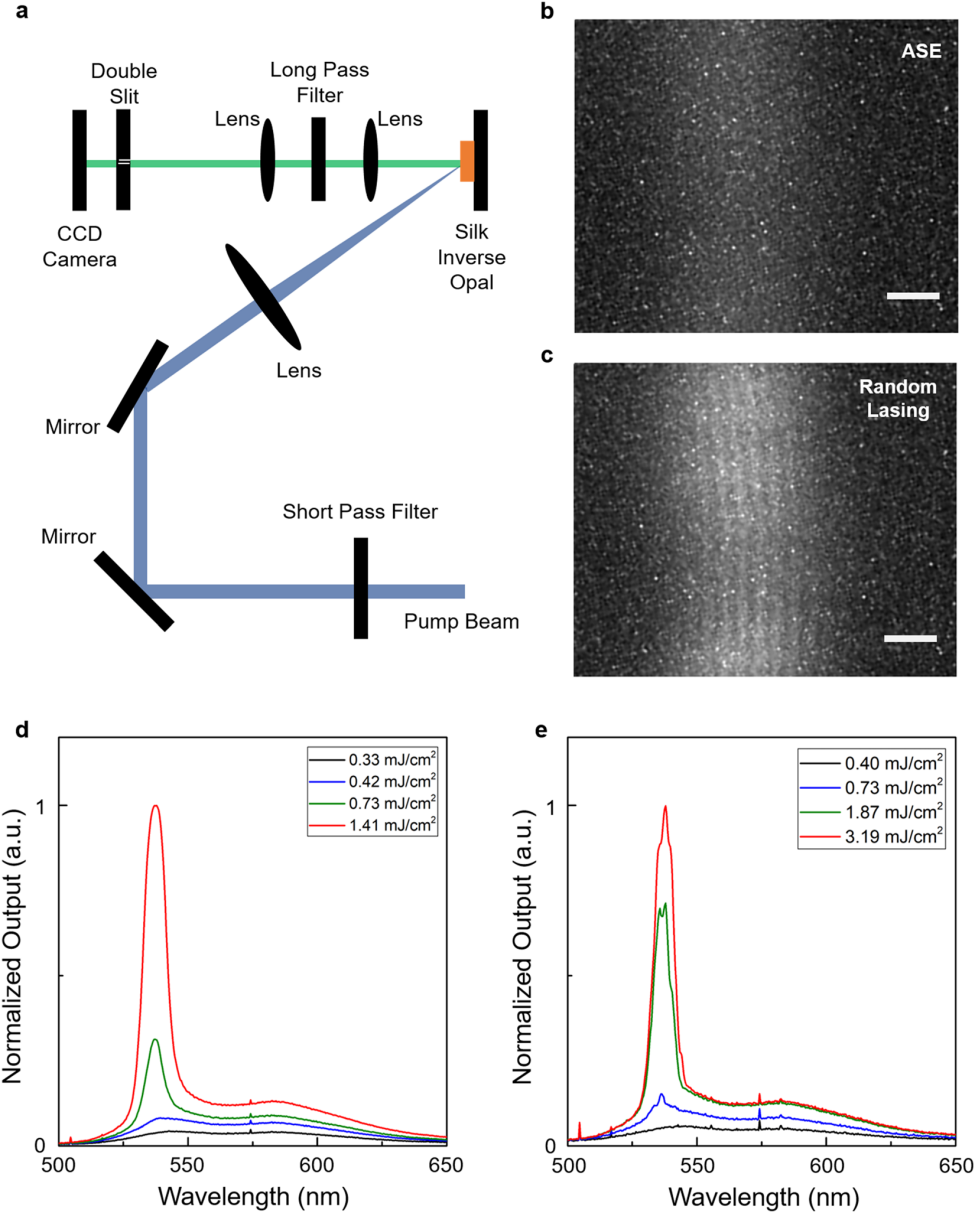
Spatial coherence of RL and ASE. (**a**) Schematic showing the experimental setup. (**b**,**c**) Far-field interference fringe images obtained from ASE (**b**) and RL (**c**) respectively. The scale bar indicates 0.1 mm. (**d**,**e**) Normalized ASE and RL spectra corresponding to (**b**,**c**).

The spatial coherence of the RL and ASE, a fundamental characteristic of lasers, was investigated by conducting Young’s double-slit experiment (Fig. 4a)^42^. A circular lens focused the RL or ASE emission to a screen with two slits (40 μm width and 250 μm separation), forming an image of the emission spot. Behind the slit, a charge-coupled device (CCD) camera was positioned at the back focal plane of a circular lens placed parallel to the slit. As shown in Fig. 4b and c, a weak interference fringe appeared for RL, comprising narrow lasing peaks on a broadband ASE band (Fig. 4e). However, no fringe appeared for ASE (Fig. 4d). The mutual coherence function, *γ*, defined as *γ* = (*I*_max_ − *I*_min_) / (*I*_max_ + *I*_min_), where *I*_max_ and *I*_min_ are the maximum and minimum intensities of the fringes, respectively, indicates the degree of coherence between two fields^42^. We obtained *γ* = 0.11 for RL, a value much smaller than that of a spatially coherent diode laser (*γ* = 0.53, *λ* = 532 nm, close to the RL emission, Supplementary Fig. 7). This measurement indicates that the spatial coherence of RL from the SIO is weak because the incoherent ASE signal overlaps and each lasing mode originates from spatially different random cavities. However, the spatial coherence can be a shred of strong evidence to distinguish the RL and ASE signals shown in silk protein scatterers.

**Figure 5:**
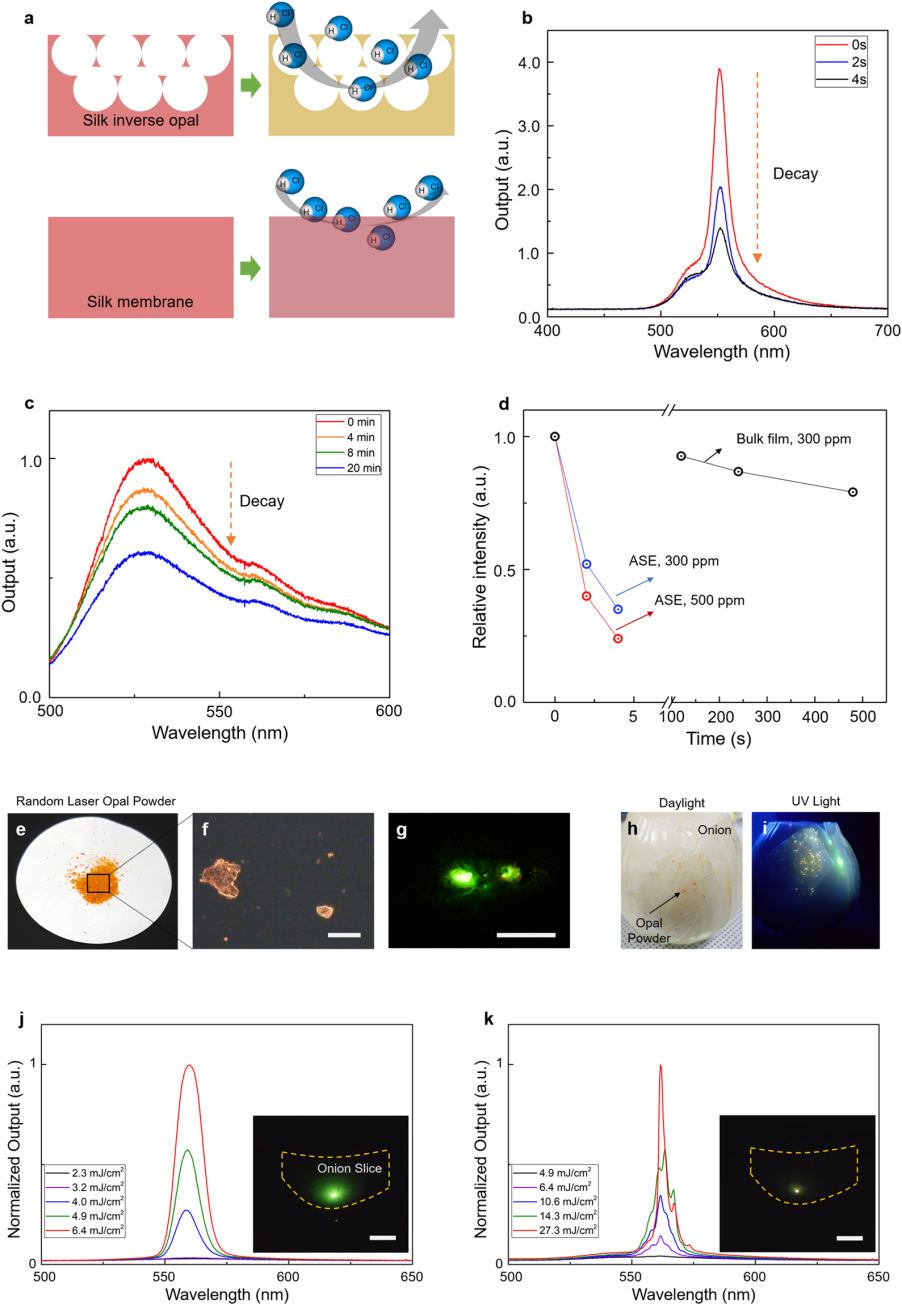
Applications of the SIO light source. (**a**) A schematic diagram to show the working principle of chemosensing by the SIO. The large surface-to-the-volume ratio and light amplification of the SIO make it possible to obtain highly efficient chemosensors. (**b**) Time-dependent ASE spectrum changes of the SIO exposed to HCl vapor with a 300-ppm concentration. (**c**) Time-dependent fluorescence changes of the bulk silk/dye film. The HCl vapor had a 300-ppm concentration. (**d**) The relative output intensities of the PL as a function of the exposure time. (**e**–**g**) Photograph and microscopic images of the SIO powder. The image in **g** shows RL from an SIO sub-mm particle under the pump fluence. Scale bars in (**f**,**g**) indicate 500 μm. (**h**,**i**) Photograph images of the SIO particles integrated under the cover tissue of an onion in daylight (**h**) and ultraviolet (UV) light (**i**). Bright PL spots of the SIO are revealed under the UV excitation. (**j**,**k**) ASE and RL spectra obtained from the integrated SIO particle. Insets are far-field CCD images showing the ASE and RL from the particle. Scale bars indicate 1 cm.

The construction of porous sensing substrates is advantageous for fluorescent chemosensing applications: their large surface areas and porous structures can facilitate the diffusion of the analyte and reduce the self-aggregation of fluorescent dyes^43,44^. Morevoer, fluorescence change detection can be further improved by applying a light-amplification scheme using photonic structures. Therefore, our porous SIO showing ASE would serve as an ideal chemosensing platform (Fig. 5a). A vapor of hydrogen chloride (HCl), which is widely used to produce organic compounds but is corrosive and harmful to humans, was chosen as an analyte to use the pH dependence of sodium fluorescein in fluorescence and in absorption. The SIO was exposed to the vaporized HCl at concentrations of 300 and 500 ppm. The exposure concentration of 300 ppm produces a 50% respiratory rate decrease after 10 min for mice (the 10 min RD_50_ value for mice)^43^. Figure 5b shows the PL spectra of the SIO exposed to 300 ppm HCl vapor. The pump power was 17.4 mJ/cm^2^; it was two times higher than the threshold value. Interestingly, 2 s was enough time to quench the ASE signal to half of its maximum value (half-period), whereas the bulk silk/dye layer (no ASE) exhibited a half-period of 20 min, which is 600 times slower (Fig. 5c). Additionally, ASE quenching was faster when the concentration of the HCl vapor was increased (Fig. 5d). It is noteworthy that the half-period shown by the SIO is four times shorter than that (8 s) of our previous study^43^, in which the fluorescent silk nanofibers of the same experimental condition are used. This result indicates that the performance of the fluorescent chemosensors can be further improved by the characteristics of the inverse opal, the large surface-to-volume ratio and fluorescence enhancement.

Full-device sizes of RL and ASE (~5 mm) are much larger than those of the resonant mode and the pumping. As such, there is plenty of room to reduce the size of the light source. As shown in Fig. 5e and f, we pulverized the SIOs to make the micron lasing particles with sizes under 800 μm. Under the pumping scheme, the SIO particles exhibited brightly luminous spots in the CCD image (Fig. 5g) and RL spectrum (Supplementary Fig. 8). The compact size and biocompatibility of the laser source offer a promising route to complementing and expanding sensing and imaging capabilities because this type of laser could be integrated with biomaterials or biosystems. As a proof-of-concept experiment, the micro SIO particles were injected underneath the outer layer of an onion (Fig. 5h). Under ultraviolet light, the particles appeared as brightly fluorescent spots through the onion tissue (Fig. 5i). Further, as shown in Fig. 5j,k, we observed the ASE and RL signals emanating from the particles underneath the tissue during pumping. The CCD images in the insets indicate that RL produces a bright spot with higher contrast, which leads to an increase in detection sensitivity for bioimaging^45^.

## Conclusion

We successfully demonstrated fully biocompatible RL and ASE light sources based on the close-packed porous structure of silk protein. The randomly generated inhomogeneity of RI distribution in SIOs yields high-Q cavities at certain spots. Narrow RLs appeared over the ASE band along with the reduction of ASE and RL thresholds. We found that when the diameter of SIO air voids was small, it was easier to get RLs and low-threshold ASE. Additionally, the bulk silk film with no photonic structure could exhibit the same ASE signal as that of the SIO, and the role of the SIO was to reduce the signal threshold by yielding random cavities. Additionally, spatial coherence, which is an inherent characteristic of lasers, was observed for RLs in Young’s double-slit experiment. The SIO was utilized as a highly efficient chemosensor to detect HCl vapor owing to the high surface-to-volume ratio and the amplification trait of the SIO. Moreover, we could make SIOs in a microparticle form suitable for bioimaging. The micro SIO particles could be integrated under the onion tissue, and they revealed the same ASE and RL signals. In view of the usefulness of lasers, we believe that the results of this paper should be very useful and timely for the development of biocompatible light sources for bioimaging and biosensing.

## Methods

### Preparation of silk fibroin solution

*Bombyx mori* coons were boiled in a solution of 0.02 M sodium carbonate (Na_2_CO_3_) for 45 min to remove sericin protein; subsequently, the remaining fibroin fibers were rinsed with distilled water and dried in air for 24 h. The dried fibroin fibers were dissolved in a 9.3 M lithium bromide solution at 60 °C for 4 h to obtain a 20 wt% aqueous fibroin solution. Then, the solution was dialyzed against distilled water using a dialysis membrane (Cellu-Sep T1, Membrane Filtration Products, MWCO 3.5 K) at room temperature for two days until the solution reached a concentration of 6 w%. The obtained solution was centrifuged twice for 20 min at −1 °C at 9,000 rpm to remove impurities and then filtered through a syringe with a pore size of 0.45 μm.

### Fabrication of SIOs

Commercially available PMMA spheres (1% concentration and dispersed in water, Phosphorex Inc.) were used to generate the opal template. An 8-μL droplet of PMMA solution was dropped onto a silicon (Si) substrate and heated at 60 °C to generate a self-assembled PMMA opal by slowly evaporating water. To break the orderness of opals, the heating temperature was increased to 115 °C to increase the speed of water evaporation. A silk solution mixed with sodium fluorescein salt (CAS: 518-47-8, Sigma-Aldrich), Rhodamine B (CAS: 81-88-9, Sigma-Aldrich), and stilbene chromophore (Stilbene 420, Exciton) were infiltrated into the PMMA opal and dried in air for 24 h. Then, the dye-doped silk film containing the PMMA opals was detached from the Si substrate and immersed in acetone for 24 h to remove the PMMA spheres. The generated SIOs with optical gain were rinsed in IPA and dried using a nitrogen gun. To make the opal powder, the SIO films were made brittle through a methanol vapor treatment for 10 h at room temperature in a closed chamber. The SIO films were then ground using a coffee grinder to an average particle size of 0.5 mm. An onion was used to obtain biological (plant) tissue. The onion’s protective layer was removed gently using a knife, and the thin tissue was cut and peeled off with care to spread the SIO powder beneath and cover it again uniformly.

### Measurement of RL and ASE from the SIO

The SIOs with optical gain were pumped using a frequency-tripled Nd:YAG laser with a 25-ps pulse width and 355-nm wavelength. The repetition rate was 2 Hz, and the beam diameter was ~3 mm. The pumping laser beam was focused onto the SIO using a circular lens to induce its optical gain. PL from the SIO was collected using a pair of circular lenses, each with a focal length of 50 mm. The focused PL spectrum was fed into the fiber of a spectrometer placed in the focal plane of a circular lens. All spectra were obtained using a fiber-optic spectrometer (USB2000, Ocean Optics) with a 1.2-nm resolution. To measure the reflection, we utilized a 1 × 2 fiber coupler that enabled the white light to be fed into the sample and the reflected signal to be captured simultaneously. The reference signal was measured using an aluminum mirror. For a comparative analysis, the SIOs with optical gain were also pumped with a nanosecond laser. We used a frequency-tripled Nd:YAG laser with a 5 ns pulse width and 2 Hz repetition rate. The pumping size was the same as that of the picosecond pumped laser.

### Spatial coherence measurement

In order to measure the spatial coherence, we focused the PL output onto a double slit with a 40-μm and 250-μm slit width and separation, respectively. (PASCO OS-8523 Slit Accessory). The fringe pattern was captured using a CCD camera (Buffered USB2.0 Color 1.3 MP, Mightex Systems) that was connected to a computer using the BUF CCD camera app. Each pixel of the image sensor was 3.75 μm × 3.75 μm.

### Preparation of vaporized HCl and concentration control

To prepare the HCl vapor environment on a parts per million scale, the HCl solution was dropped using a micropipette in a beaker. After the beaker was sealed, HCl fully vaporized for 2 h. For a concentration of 1 ppm, the ratio of the volume of the HCl vapor to the beaker volume was adjusted to 1 μL/L. By evaporating 1.63 μg of HCl solution, 1 μL of HCl vapor could be obtained, as estimated from the ratio of molar mass to molar volume at room temperature.

## Supplementary information

Supplementary Information
