# Supplementary material for: Random lasing and amplified spontaneous emission from silk inverse opals: Optical gain enhancement via protein scatterers
Source: Sci Rep. 2019 Nov 7;9:16266. doi: 10.1038/s41598-019-52706-4 (PMC6838073; doi:10.1038/s41598-019-52706-4)
Supplement: Supplementary file 1 — Supplementary Information [file 41598_2019_52706_MOESM1_ESM.docx]

Supplementary Information for

Random lasing and amplified spontaneous emission from silk inverse opals: optical gain enhancement via protein scatterers

Muhammad Umar^1,†^, Kyungtaek Min^1,2,†^, Sookyoung Kim^1^, and Sunghwan Kim^1,3,*^

^1^Department of Energy Systems Research, Ajou University, Suwon 16499, Republic of Korea

^2^Department of Nano-Optical Engineering, Korea Polytechnic University, Siheung 15073, Republic of Korea

^3^Department of Physics, Ajou University, Suwon 16499, Republic of Korea

^*^To whom correspondence should be addressed. E-mail: [sunghwankim@ajou.ac.kr](mailto:sunghwankim@ajou.ac.kr)

^†^These authors equally contributed to this work.


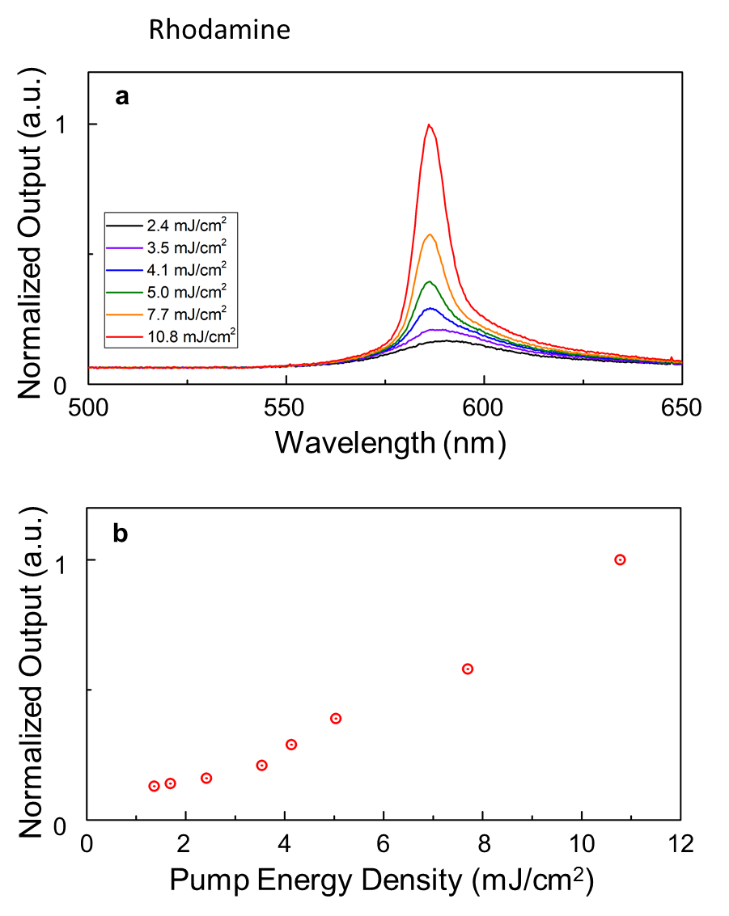


**Supplementary Figure 1 | Emission from the silk/Rhodamine B (RhB) film**. **a,** Normalized emission spectra of the silk film containing the RhB gain. The spectra display the transition from fluorescence to ASE as the pump energy density is increased. **b,** Light-in versus LL curve exhibiting the output intensities of the ASE peaks as functions of the pump energy density.


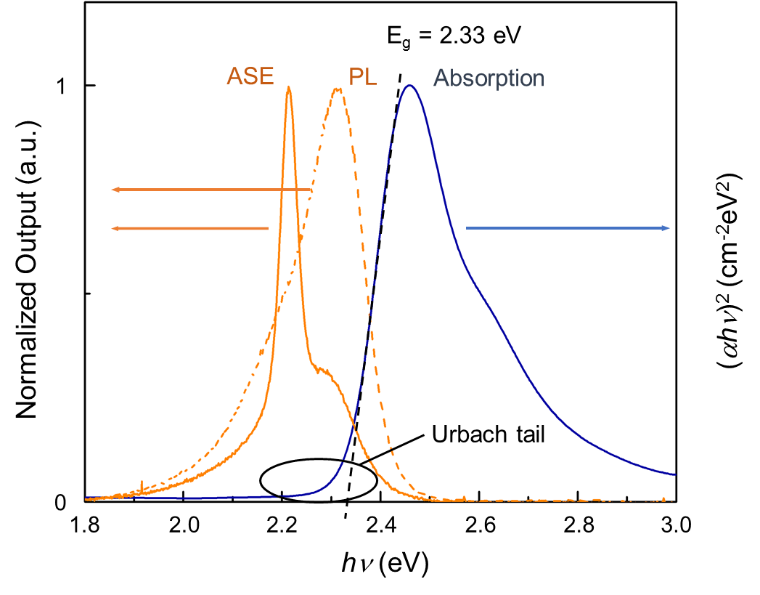


**Supplementary Figure 2 | Investigation of the band gap and tail of the sodium fluorescein dye.** The absorption spectrum, replotted fluorescence, and ASE spectrum, from sodium fluorescein using Tauc's model. Sodium fluorescein shows the direct band gap at approximately 2.33 eV (dashed line) at the point where the Ubrach tail (circled) and the ASE spectral maximum coincide.


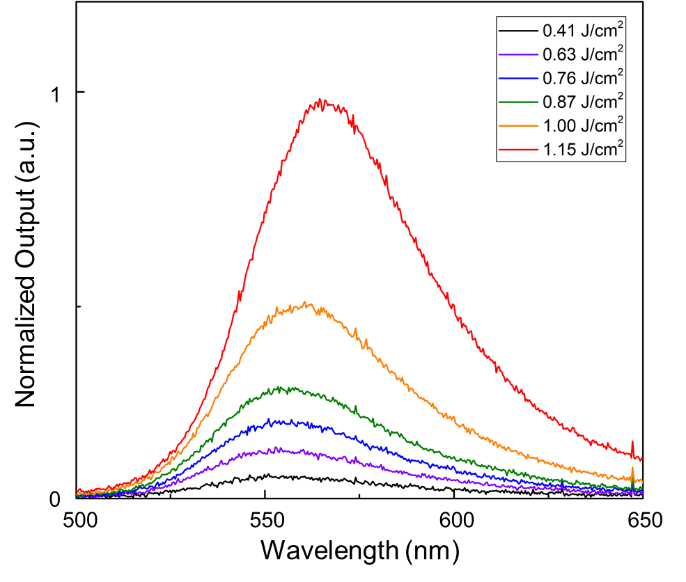


**Supplementary Figure 3 | PL emission from SIO under ns pulses.** The normalized emission spectra from the SIO with sodium fluorescein when pumped with 5-ns pulses at 355 nm. The laser repetition rate was 2 Hz, and the focused beam diameter was 0.25 mm. All the pumping laser parameters except the pulse width were kept constant.

**Losing the high-Q random cavity by decreasing the RI contrast**

The RI contrast between silk (*n*_silk_ = 1.54) and air (*n*_air_ = 1) in the SIO is significant enough to yield high-Q random cavities. Lowering the RI contrast can reduce the Q of the random cavities owing to diffusive reflection. Figures 4a and c show the appearance and reappearance of the RL spectrum from the SIO when the sample was in the air and was dried after immersion in IPA owing to the high-Q cavities formed by the high silk-air RI contrast (*Δn* = 0.54). Figure 4b indicates the transition from the RL to ASE emission when the SIO was immersed in IPA. The transition of lasing to ASE was due to the reduced RI contrast (*Δn* = 0.16) by filling air voids with IPA to yield low-Q random cavities. Additionally, the reduction in the RI contrast increased the threshold of ASE because of the increased mean free path length (Fig. 4d).


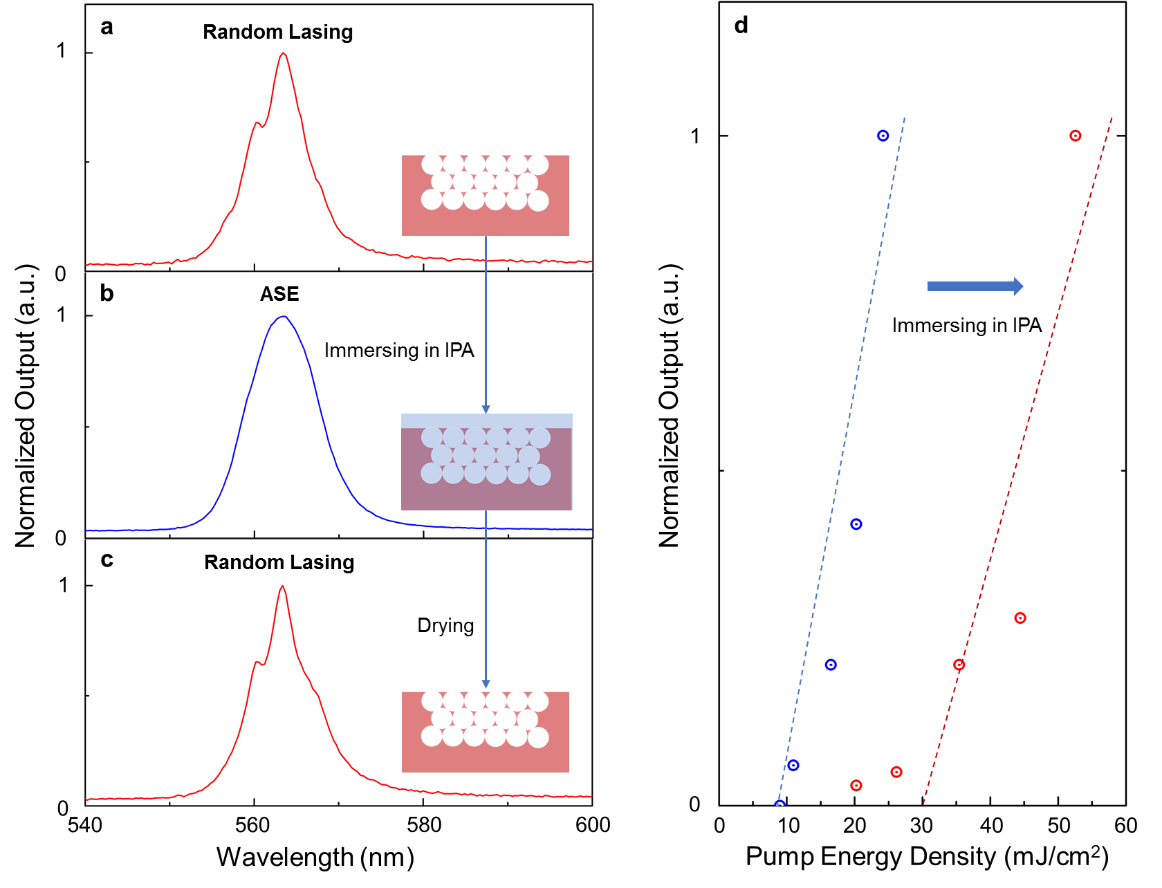


**Supplementary Figure 4 | RI contrast effect on emission properties of the SIO.** Normalized output spectra from the optically pumped SIO in air and immersed in IPA. **a-c,** Emission spectra displaying the transition between RL (air environment) in **a** and **c** and ASE (IPA environment) in **b**. **d,** LL curve of the ASE maxima obtained from the SIO in the air (blue) and immersed in IPA (red).


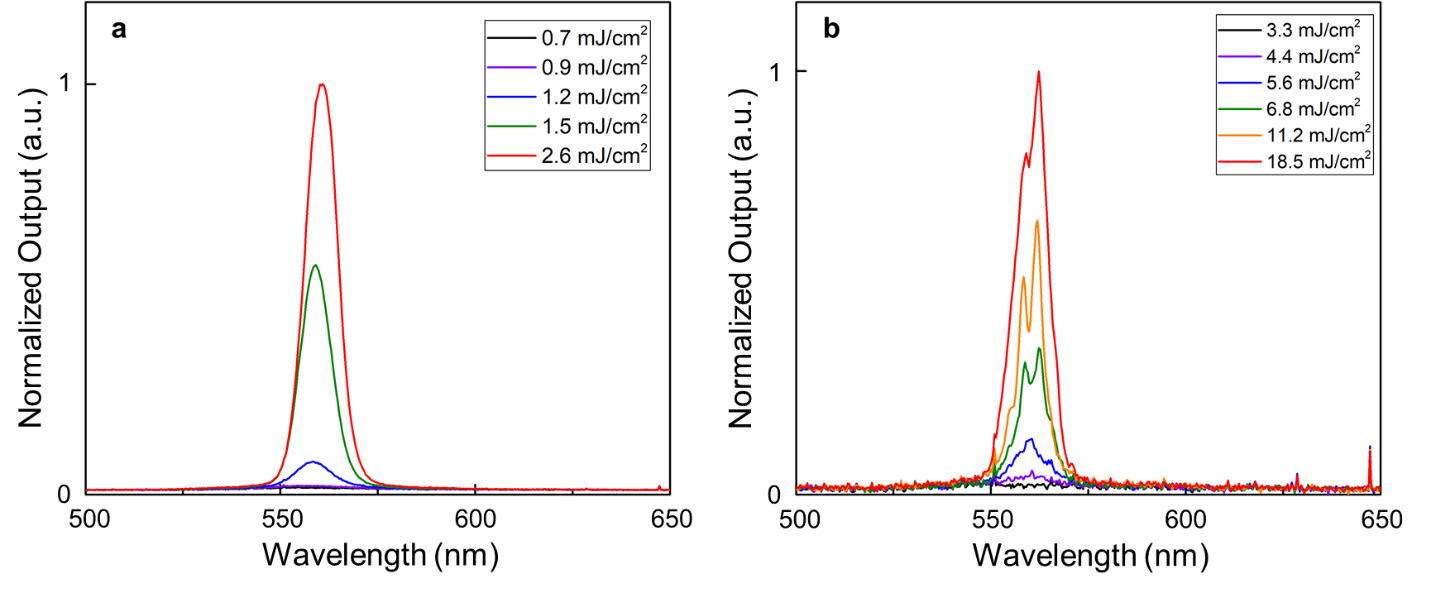


**Supplementary Figure 5 | ASE and RL spectra from the SIO with shorter scatterers. a,b,** ASE and RL spectra from the SIO with 100-nm air voids under different optical pumping.

**ASE and RL from SIOs with different organic dyes**

In the healthcare systems for diagnoses and imaging, using various biological tracers is essential to enable the application of the systems to various environments. Silk, a natural protein, is a promising material for incorporating a variety of biological tracers in silk-based bio-optical devices. Stilbene chromophores could be useful as tracers for blue emission and produce high optical gain when incorporated into the silk matrix. In addition, the scattering in the SIO is significant enough to induce a strong gain enhancement for blue light, which has a shorter wavelength than that of sodium fluorescein. The optical gain enhancement under the increasing pump energy density reveals a rising ASE and RL from the SIO mixed with a stilbene dye, as shown in Figs. 5a and b.


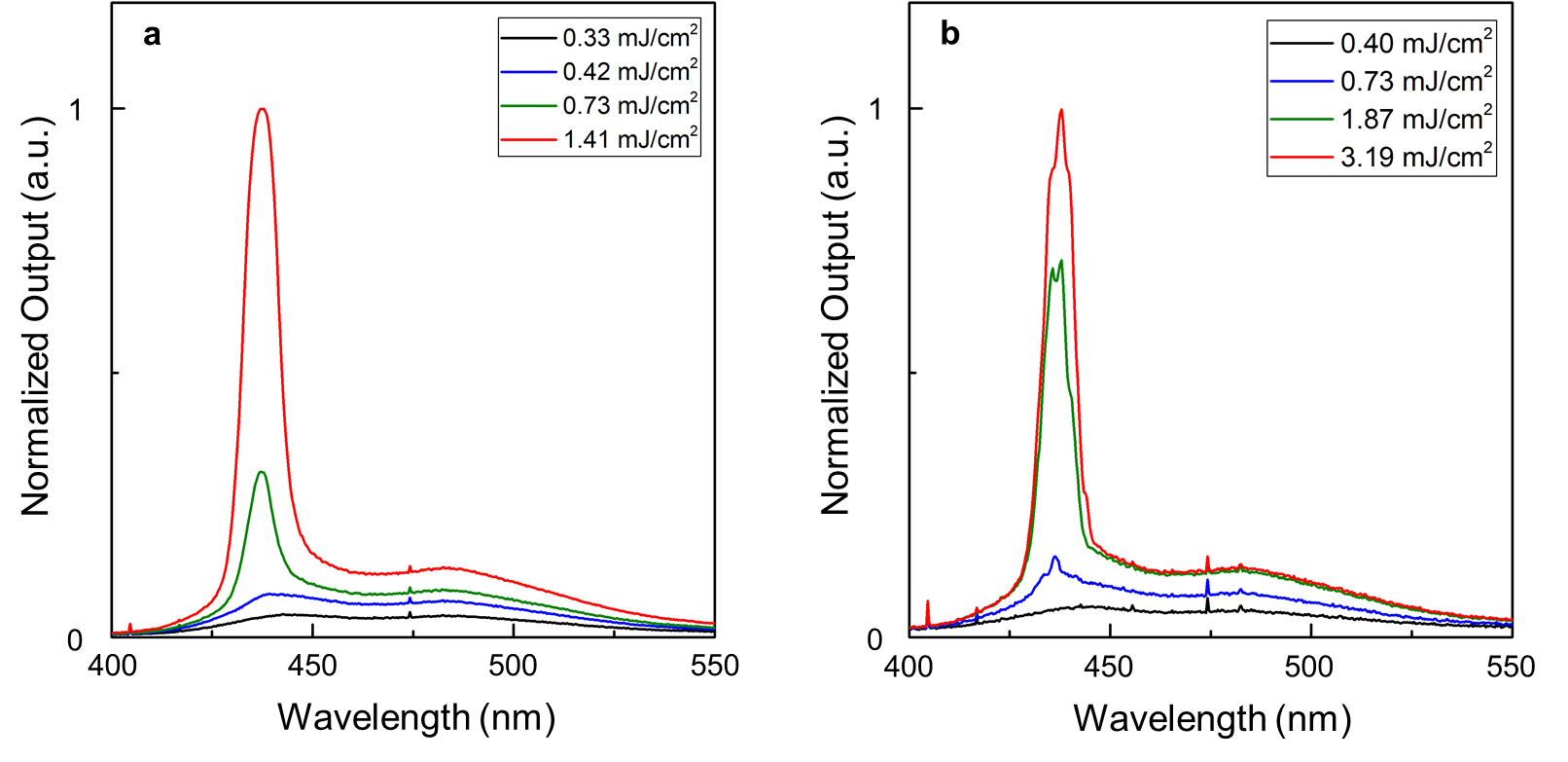


**Supplementary Figure 6 | Optical gain enhancement of the stilbene-mixed SIO.** ASE spectra in **a** and RL spectra in **b** obtained from the SIO mixed with stilbene chromophores.


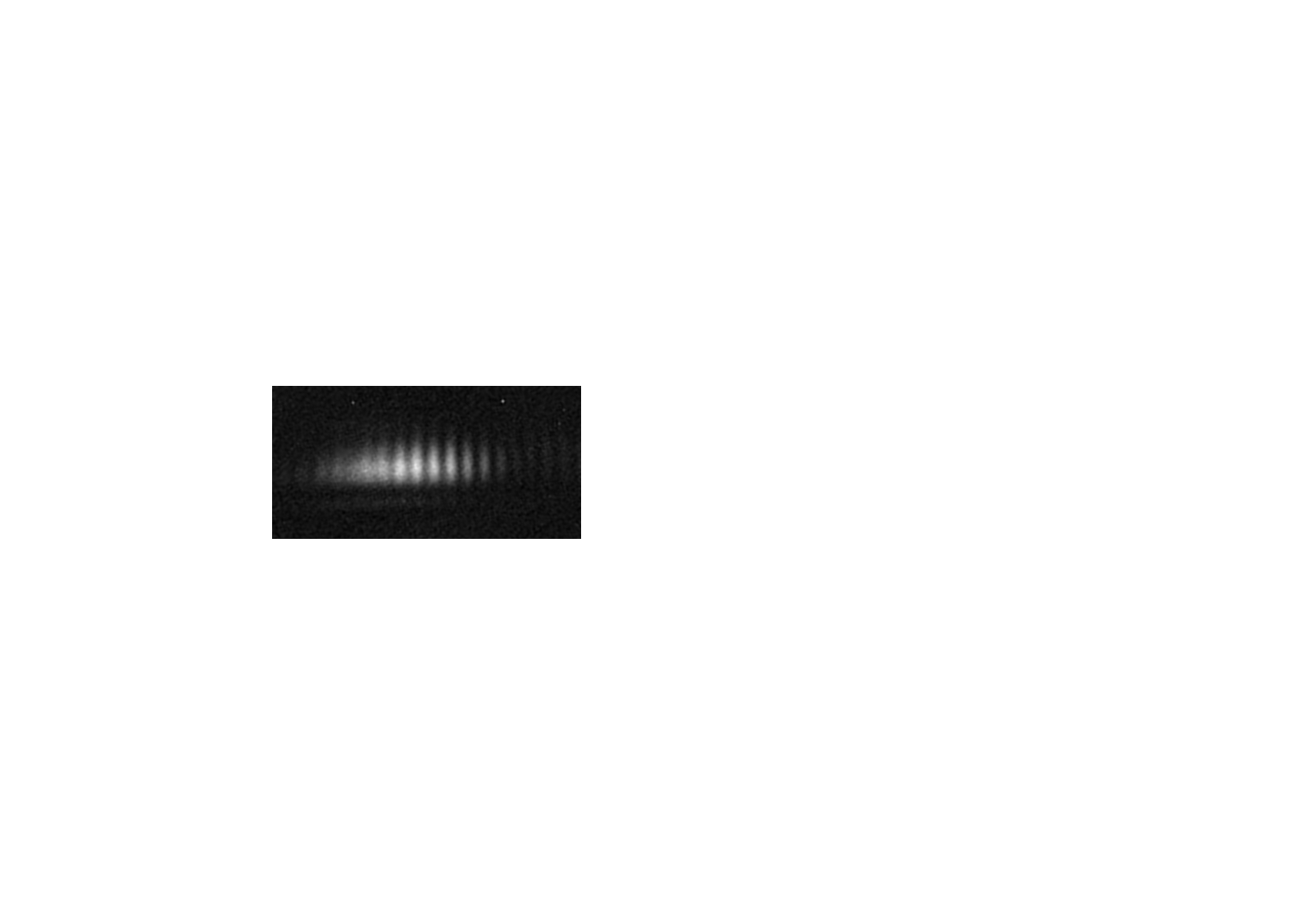


**Supplementary Figure 7 | Spatial coherence of a diode laser.** A clear far-field interference pattern obtained using a diode laser with a 532-nm wavelength indicates that the spatial coherence depends on the number of modes of the incident laser light.


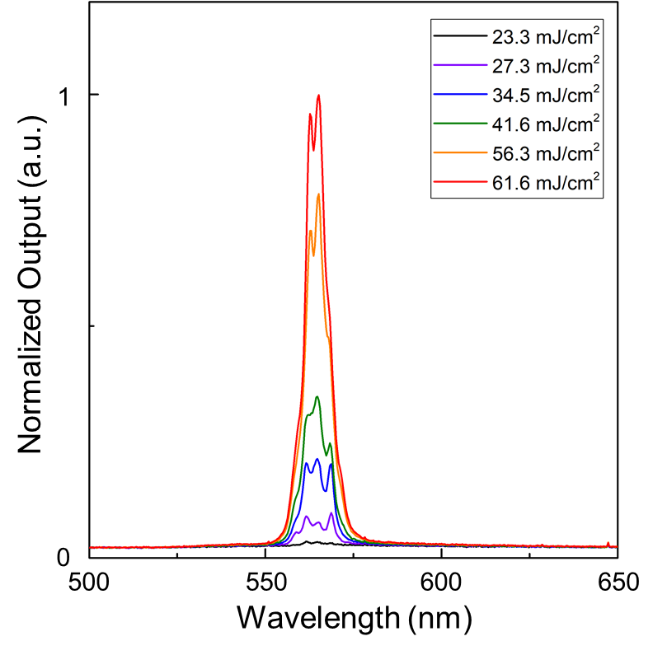


**Supplementary Figure 8 | RL from an SIO micron particle.** For a compact and coherent light source, we generated the powder form of the SIO with an average size of 800 μm that could exhibit emerging RL peaks as the pump energy density is increased.
